# Bibliography

**Published:** 1902-01-15

**Authors:** 


### BIBLIOGRAPHY.

Questions and Answers for Dental Students is a book of 500 pages giving classified questions and answers on all the subjects usually taught in the dental college course. The work is from the pen of Prof. F. J. H. Gorgas, dean of the dental department of the University of Maryland, and the well known author of several dental text books. It would be an almost impracticable thing for one to undertake to make a classification that would please every teacher, and we doubt not many teachers will feel that they could have arranged the questions more systematically in their respective subjects, but we think Prof. Gorgas has done well indeed to cover so many subjects and do it as well as he has succeeded in doing in this work. Most every topic treated, seems to have been more or less slighted when compared with actual class-room quiz work, but we do not understand that this is the intention of the work. It will doubtless serve its purpose in crystalizing the reading of students and such as are preparing to take State board or similar examinations. The work is well done. We note some erroneous statements, as for instance on page 99 the definition of pharmacology is all wrong, it is a definition of pharmacy, and again on page 115, in answer to the question as to how crystal gold is made, the answer is "by electrolysis," which is not correct. The important subject of bacteriology is given only five pages of questions and answers, whilst the subject of dental metallurgy has forty pages. The book is well printed and bound and is another evidence of the capacity and worth of the well known publishing house of Blakistone's Son & Co., of Philadelphia.

---

Principles and Practice of Operative Dentistry, by John S. Marshall, is a new and comprehensive work on operative dentistry, just from the press of the J. B. Lippincott Co., of Philadelphia. We are somewhat surprised to have another work on this subject at this time and also from this source, as we have had several new works on this subject from teachers of this subject and we had felt that the field was tolerably well occupied. Dr. Marshall is a good writer and a teacher of experience and he writes for the practitioner as well as the student, and in some respects his book may be looked upon as a treatise rather than a class book. It is a large work of over six hundred pages and abundantly as well as beautifully illustrated. A large part of the illustrations are from microscopic slides and are well brought out in the printing. There are in all 732 illustrations and yet this feature does not appear to be overdone, The work gives a comprehensive treatment of the anatomy, physiology and pathology as a basis for subsequent treatment. It then takes up the prophylactic and surgical treatment, covering every feature in a very thorough and satisfactory manner. The last twenty chapters are largely devoted to the pathologic and therapeutic phases of operative dentistry and might very well be considered as a separate subject, did not much of the treatment involve operative or instrumental procedures, or have a very intimate relationship. There is little to adversely criticise in the work, because there is so much to praise. It is a work which will have a long life and meet the needs of the practitioner better than any other work. While Dr. Marshall has availed himself of the valuable services of others in its preparation, the work is a consistent and sequential treatment of operative practice upon a sound scientific basis and must command the confidence and respect of the best practitioners. We regret we have not space for a more detailed reference. It is a worthy book and the profession is under obligations to its author.

